# The Idea of Precaution: Ethical Requirements for the Regulation of New Biotechnologies in the Environmental Field

**DOI:** 10.3389/fpls.2018.01868

**Published:** 2018-12-21

**Authors:** Klaus Peter Rippe, Ariane Willemsen

**Affiliations:** ^1^Pädagogische Hochschule Karlsruhe, Karlsruhe, Germany; ^2^Eidgenössische Ethikkommission für die Biotechnologie im Ausserhumanbereich, Bern, Switzerland

**Keywords:** precaution, ethics, new technologies, biotechnology, regulation, risk evaluation, precautionary measures

## Abstract

The rapid emergence of new biotechnologies for selectively altering genetic material—so-called genome editing—has sparked public controversy about how their development and application in the environmental fields are to be regulated. Since the use of these new technologies harbors not only considerable potential but also risks of serious damage whose occurrence is uncertain due to their application in complex environmental systems, many national and international legal authorities are currently adhering to policies of precaution. According to critics, however, precautionary measures and the legal principle of precaution on which they are based are unduly restrictive in the case of the new biotechnologies, hindering advancements in both research and various fields of application. At the same time, legal notions of precaution are highly ambiguous within and across different national and international formulations, thereby further complicating the controversy about their implications. This paper goes beyond the concept of precaution as found in environmental law by examining the *ethical* significance and the *ethical* justification of precautionary measures in the environmental field. In particular, it clarifies the criterion of potential damage, disambiguates different types of epistemic bases in precaution decisions, and considers the relevance and implications of different ethical risk theories as to their response to epistemic uncertainty and vagueness. The two main conclusions are that, first, irrespective of the ethical risk theory embraced, there is an ethical obligation to take precautionary measures whenever serious damage is possible and the probability of damage occurring epistemically uncertain or vague. Regarding the risk assessment, it is argued that the burden of proof lies not with those who fear the occurrence of serious environmental damage. Rather, it is up to those whose actions give rise to such fears to demonstrate that serious damage is extremely improbable or scientifically absurd. Second, the moral responsibility to determine precaution situations and to specify appropriate precautionary measures is attributed not only to state authorities but also to industrial players as well as research communities. Based on these two conclusions, recommendations are given as to how the precautionary principle should be incorporated in political and legal decision-making.

## Introduction

The rapid development of new techniques which allow us to selectively alter genetic material, and are thus termed *genome editing*^1^, has sparked public discussion about how such biotechnologies are to be regulated. On the one hand, the new biotechnologies appear to harbor considerable potential for research and for many areas of application. In the mosquito that spreads malaria, for example, it is now feasible to produce so-called gene drives^2^ which could be deployed to diminish disease carrier populations (cf. for example Hammond et al., 2016). On the other hand, due to their application in complex environmental systems in which the occurrence of serious damage is typically uncertain, many national and international legal authorities are currently adhering to policies of precaution. In Switzerland, for example, authorities currently assume that all so-called new procedures are genetic engineering procedures, and therefore fall under previously established genetic engineering regulations that require relatively strict authorization procedures. According to critics, however, precautionary measures and the legal principle of precaution on which they are based are unduly restrictive because the intended alterations to the genome are either no longer detectable in the product or may well be the result of natural mutations.

Legal notions of precaution are highly ambiguous within and across different national and international formulations, thereby further complicating the controversy about their implications. This paper goes beyond the concept of precaution as found in environmental law by examining the *ethical* significance and the *ethical* justification of precautionary measures in the environmental field^3^. It shows how precaution is a (morally) significant action-guiding principle in the regulation of new biotechnologies, and describes the broader conditions and (moral) responsibilities across a wide range of actors for precautionary measures to have their desired effect. In doing so, the scope of the considerations and arguments presented here is limited in at least two respects. First, the main aim of this paper is to show how the idea of precaution bears ethical relevance in the regulation of new environmental (bio-) technologies, thereby foregoing any attempt to offer a full (philosophical) defense of the principle. Note that in bioethical debates in particular, ideas about the (moral) value of precaution are only beginning to be developed (cf. for this assessment of the debate Munthe, 2015). The paper contributes to the debate within environmental politics and, hence, is intended primarily for an interdisciplinary, policy-oriented audience. Second, since the ethical analysis of the idea of precaution focuses on the context of environmental (bio-) technology and decision-making, it is up to further discussion whether its conclusions apply also to other areas in which reference to precaution are increasingly made, such as in medical health care or climate policy.

As a starting point of the ethical analysis, this paper will draw on both the concept of precaution as it is originally found in environmental law as well as on the everyday understanding of precaution and precautionary measures (section Precaution as a Concept in Environmental Law and the Term “precaution” in Specialist and General Parlance). Since, however, neither environmental law nor everyday language provide an answer to the question of how a precautionary approach in the environmental field can be *ethically justified*, the paper will look more closely at whether, and to what extent, legal and day-to-day criteria for introducing precautionary measures are also relevant from an ethical point of view. In particular, it clarifies the criterion of potential damage, disambiguates different types of epistemic bases in precautionary decision-making, and considers the relevance and implications of different ethical theories of risk as to their response to epistemic uncertainty and vagueness (section The Ethical Idea of Precaution). The two main conclusions are that, first, irrespective of the ethical theory of risk embraced, there is an ethical obligation to take precautionary measures if serious damage is possible, and if the probability of damage occurring is epistemically uncertain or vague. Regarding the risk assessment, it is argued that the burden of proof lies not with those who fear the occurrence of serious environmental damage. Rather, it is up to those whose actions give rise to such fears to demonstrate that serious damage is extremely improbable or scientifically absurd. Second, the moral responsibility to determine situations of precaution and to specify appropriate precautionary measures is attributed not only to state authorities but also to industrial players as well as research communities (section Precautionary Obligations). Based on these two conclusions, recommendations are given as to how the precautionary principle should be incorporated in political and legal decision-making (section Recommendations).

## Precaution as a Concept in Environmental Law and the Term “Precaution” in Specialist and General Parlance

### Precaution as a Concept in Environmental Law

The classic legal model to protect the public from damage comes from hazard prevention. Toward the end of the twentieth century, the conviction became established in environmental policy that in certain situations it is not enough to react only when a threat is imminent or when a threat of damage is certain. Protective measures should also be taken—as a precautionary measure—even if it is not yet known whether and with what probability such damage will occur. This idea of precaution was increasingly included in the discussion on environmental law and has subsequently become firmly established in various legal documents at national and international level.

An important milestone in the establishment of the principle of precaution in law at international level was the 1992 Declaration of the United Nations Conference on Environment and Development of Rio de Janeiro (Rio Declaration)^4^. Principle 15 formulates the idea of precaution: “In order to protect the environment, the precautionary approach shall be widely applied by States according to their capabilities. Where there are threats of serious or irreversible damage, lack of full scientific certainty shall not be used as a reason for postponing cost-effective measures to prevent environmental degradation.” The European Commission addressed the concept of precaution in its Communication in the year 2000^5^. In the meantime, it has become an established regulatory principle of environmental legislation. Precaution is applied when preliminary risk assessment indicates that there are reasonable grounds for concern that something has a potentially dangerous impact on the environment, human, animal or plant health, even when scientific evidence is insufficient, inconclusive or uncertain^6^. Swiss environmental legislation also addresses the issue of precaution. The Federal Constitution requires that damage or nuisance be avoided^7^. The Environmental Protection Act^8^ and the Gene Technology Act^9^ state that such damaging and disturbing impacts are to be limited at an early stage.

These documents differ in the way in which they formulate the concept of precaution. Whereas the European Commission talks of the *precautionary principle* in its communication, the Rio Declaration uses the term *precautionary approach* in the English version*, Vorsorgegrundsatz (engl. precautionary policy/principle*) in German, and *mesure de précaution (engl. precautionary measure)* in French. The Swiss formulations talk of avoiding damage and nuisance to the environment. The Environmental Protection Act and the Gene Technology Act state that such impact is to be limited at an *early* stage.

It is conceivable that different things are intended with these different formulations, and that the idea of precaution may not involve one principle or approach, but a whole array of different principles or approaches (cf. Hartzell-Nichols, 2013). Alternatively, it may be that the idea of precaution is formulated differently in varying contexts, but that the same set of legal instruments is ultimately established. In any case, it can be said that all formulations have a common core (cf. Gardiner, 2006). Precautions should be taken to avoid damage when two criteria are met: (1) it is feared that damage (of a certain extent) may occur and (2) knowledge about the probability of such damage is restricted. According to the Rio Declaration, the possible damage must be serious or irreversible and the restricted knowledge must constitute scientific uncertainty. In the European Commission's communication, the severity of the damage is not qualified, and a preliminary scientific risk assessment must give cause for concern.

The formulations in Swiss law differ from the internationally established understanding of precaution in a variety of ways. They state that not only harmful effects but also nuisances must be prevented, and the criterion of restricted knowledge is not explicitly mentioned. Furthermore, there is no mention of scientific uncertainty or of preliminary scientific risk assessment^10^. It may be said that the idea of precaution, as it has been discussed since the Rio Declaration in 1992, only finds expression in Swiss environmental law in individual pieces of legislation such as the Gene Technology Act.

This paper aims to respond to the core requirement of all these formulations, namely the need to react to the fear of potential harmful effects, and to the question of how such a requirement and the resulting obligations can be ethically justified.

### Precaution and Prevention

In German, the terms *Vorsorge* (precaution) and *Prävention* (prevention) are widely used synonymously, both in technical jargon and in everyday language. German-language legal texts sometimes use the term *Prävention* in the context of precaution. In French and Italian, the two terms are also generally used synonymously in everyday usage. On the other hand, specialist literature in these two languages distinguishes clearly between *précaution*/*precauzione* and *prévention/prevenzione*: if the probability of occurrence of damage is known, the term used is *prévention*/*prevenzione*; if, however, the probability of damage occurring is uncertain, the term *précaution/precauzione* is employed^11^. As this paper examines the question of how uncertainty is to be addressed, it also looks at the ongoing discussion in French and Italian specialist language of *précaution/precauzione*, respectively.

### The Broad Understanding of Precaution in Everyday Language and the Narrow Understanding of the Precautionary Requirement in Environmental Law

In contrast to the (international) concept of environmental law, in our day-to-day lives we not only invoke precautionary measures when there is a threat of serious, major or irreversible damage. Rather, we typically consider precautions and corresponding measures even in response to minor harmful scenarios: for example, if unsettled weather is forecast and—as a precautionary measure—we take along a raincoat. Moreover, according to this general colloquial understanding, we also speak of precaution when a situation that is to be assessed negatively might occur not only possibly, but with a very high probability, or even with a probability bordering on certainty. In everyday language, in other words, we use the term “precaution” for situations in which one could (also) speak of prevention. Saving for an old-age pension provides an example of this: even if a person does not know with certainty whether they will reach retirement age, it is rational to take precautionary measures for the loss of income associated with retirement. Or if a single parent knows there is a probability bordering on certainty that they will soon die, and if they can prevent or alleviate some of the negative consequences for the family members left behind after their death, they have a moral duty to take appropriate precautions. Similarly, if a person must assume with near certainty that their behavior will result in others becoming infected with a dangerous disease, she is obliged to take (preventive) measures.

This broad and general understanding of precaution thus means preparing based on one's own or another's assessment of the risk to avoid or alleviate harmful effects that could occur as a result of subjective or objective assumptions of probability. Precautionary measures are decided on this basis. Leaving aside the question of moral duty toward oneself, precaution can also be generally understood as an *ethical duty* either to protect others from harm or to avoid risks of harm that we inflict upon others.

However, even with this general understanding of precaution, it may well be that it also incorporates the idea that possible harmful effects must be of a certain quality in order to justify an obligation to take precautionary measures. On the other hand, according to this broad understanding, there is no precautionary situation and therefore no obligation to take precautionary measures if there is no indication that harmful effects may ensue. This does not mean that no harmful effects can occur; only that it is at present unknown that something is unknown. Moreover, one is not required to be aware of not knowing. This means that even in the everyday understanding of precaution, no one has a moral duty to take precautionary measures against previously unobserved harmful effects or harmful effects that have not yet been observed or deemed possible.

In environmental law, the understanding of precaution is somewhat narrower. Here, the demand for precaution arises in the face of the fact that the scope of our knowledge is restricted. Either the understanding of precaution in environmental law thus refers to a special case of the everyday concept of precaution, or it designates an ethical principle that is distinct from the broad and general understanding of precaution described above.

A look at both environmental law and everyday language serves as a first approach to the possible meaning(s) of the precautionary idea and provides indications as to which situations can call for precautionary measures. However, neither environmental law nor everyday language can provide an answer to the questions of how a precautionary obligation can be *ethically justified*, who bears an obligation, and what this obligation consists in. Thus in the following, we will examine whether and to what extent the criteria for introducing precautionary measures found in the law are also relevant from an ethical point of view, and whether there may be grounds for further obligations beyond these legal criteria. This analysis takes the criteria in environmental law as a starting point, but then continues separately from the legal considerations. A link to the law is re-established after the conclusion of the ethical analysis, in order to reflect these considerations in existing law and to clarify possible need for action.

## The Ethical Idea of Precaution

### The Criterion of Potential Damage

The core idea of precaution is that certain harmful effects should not occur and that one should take measures to prevent or limit them whenever possible. In formulations in internationally relevant environmental legal texts, the duty to take precautionary measures does not relate to all harmful effects, but only to those of a certain quality. According to the Rio Declaration, the duty to protect in the sense of precaution only extends to potentially serious or irreversible damage to human health and the environment. The communication of the European Commission accords this particular quality to damage to the environment and human, animal and plant health if it exceeds a certain level. This damage can be understood to constitute the impairment of legally defined objects of protection or protection goals. Besides damage to health and the environment, there may be other (also serious) effects of an economic nature. However, under international environmental law there seems to be no precautionary obligation to protect against such effects.

For an ethical examination of the idea of precaution, the criterion of potential damage raises two main questions. On the one hand, it may be asked how an obligation to precaution, which relates to damage that is not certain but possible to occur (in the sense that there are plausible grounds to fear its occurrence) can be justified. On the other hand, we must establish what justifies the restriction of these obligations to a particular type or quality of possible damage. In order to answer these questions, we must first look more closely at what constitutes damage.

### What Constitutes Damage and Who or What Can Suffer Damage?

A plausible definition of damage is a change that must be judged to be negative. It is irrelevant who causes the damage. The damage is the same whether humans cause it or it is the result of natural forces.

Damage is morally relevant when it affects entities that themselves have moral value. Who or what these entities are depends on the position held in (environmental) ethics. Here, we restrict ourselves to a selection of four options that are most frequently referred to:

- Anthropocentric positions place humans at the center in the sense that only humans count morally for their own sake. Only humans, therefore, can suffer damage for their own sake. According to this position, serious damage to animals, plants or the environment is morally relevant only insofar as it affects humans and, correspondingly, these entities are merely of instrumental or relational value to them.
- Pathocentric positions place a living being's sentience and ability to feel pain as the main criterion to determine whether it can suffer damage. A living being can suffer damage provided it has some form of inner experience or if it can experience something as good or bad.
- Biocentric positions consider all living beings to have moral value for their own sake. For these positions, sentience is not a prerequisite for a being's inherent value. There are two main biocentric approaches. According to one approach, living beings have inherent value and can therefore be damaged, because being alive has value for its own sake. According to the second approach, living beings can be damaged because as bearers of a good life they have a good of their own. This second approach assumes that living beings have, so to speak, an inscribed aim specific to their species.
- Ecocentric positions focus not just on living beings but the whole of nature as a comprehensive, complex interaction between entities. If we interpret this position holistically, collective entities such as ecosystems, biotopes, species and populations, nature, the earth or even the whole universe have inherent value. For advocates of an individualistic reading of this position, all individual beings that are part of nature count morally for their own sake, both living beings and non-living beings such as lakes, mountains or landscapes. All of these collective or individual entities can be harmed.

Depending on the position held in environmental ethics, different entities will be among those beings that can be harmed for their own sake. This, however, does not yet tell us how much the damage caused to such an entity counts. There are essentially two answers to this question. The egalitarian position assumes that equal damage caused to any entity that can be harmed must be assessed equally and unequal damage differently. According to a hierarchical position, all entities that can be harmed should be considered. However, as not all entities have equal value, the damage caused to (hierarchically) different entities counts differently. Either the nature of the species counts, so that interests, such as those of humans, are weighted more highly than equal interests of other entities. Or, the complexity of characteristics counts, and the more similar the characteristics to those of humans in terms of their complexity, the higher the harmful effects are weighted^12^.

### The Ethical Significance of Qualifying Damage in the Context of Precaution

In contrast to the broad everyday understanding of precaution, according to which precautionary measures should be taken against even the slightest of harmful effects, in a narrower understanding of the concept, as it is formulated in environmental law, the quality of the damage plays an important role^13^.

One reason for restricting precautionary obligation in environmental law to a particular type of damage may lie in the fact that the State is under an obligation to intervene in basic rights, in particular rights of freedom. Any intervention in basic rights requires justification. Another or additional reason could be that at international level only a qualified type of damage could be agreed on for political reasons.

For the purposes of this discussion, irrespective of any possible politically motivated reason for limiting the concept of precaution to certain types of damage, we will look at the normative question (which is also relevant for a legal justification) as to how far such a limitation can be ethically justified. Two main positions can be distinguished regarding the normative meaning of damage. The first position assumes that certain types of damage cannot be compared with others; the second assumes that all types of damage can and may be compared:

1. The first position assumes that certain types of damage represent a negative outcome of a type that cannot be compared and therefore not be weighted against other negative outcomes. These types of damage thus form their own normative category. If it is conceivable that damage of this type could occur in a certain situation, there is either a duty to refrain from action or a requirement to act (e.g., to generate knowledge as a prerequisite for a risk assessment). Damage of this kind must always be avoided. Even if the probability of damage occurring is extremely slight, it is the extent of the potential damage that is of relevance. For if risk is a function of damage and probability of occurrence, and if the negative outcome is astronomically severe damage, then even the smallest probability of occurrence would result in an immeasurably great and therefore impermissible risk. The key question to be asked in this position is: what constitutes incomparably severe damage?
  There are two variations of this first position. According to the *first variation*, the *physical* destruction of the whole of humanity would constitute incomparably severe damage, whereas according to the *second variation*, it is the *cultural* destruction of humanity, which meets the criterion of incomparably severe damage. Even if, following a catastrophic nuclear event, a large number of people could continue to live biologically, but not in a way that constitutes the cultural nature of humans, then according to the second variation, this would constitute incomparably severe damage and hence an evil that must be prevented at all costs. It is inadmissible to weigh up such damage against other interests.
  Advocates of both variations of this first position agree with the second position set out below that a weighing of interests is admissible with regard to all other interests.
2. According to the second position, no damage can be of a quality that does not allow comparison with other types of damage. If different instances of damage can only be distinguished by their extent, it can still be assumed that only once the damage reaches a certain extent is it necessary to act (which may also mean refraining from doing anything). This would then give us a conception of a threshold. Only when the possible damage reaches a certain level does precaution come into play in situations where knowledge is limited, and the obligation arises to take measures to prevent damage of this magnitude. If the possible damage does not reach this threshold, precautionary measures are not required, even in situations of scientific uncertainty. The key question to be asked in this position is: when is this threshold reached?
  A *variant* of this second position also includes small-scale possible damage in the consideration of precaution. According to this position, requiring precautionary measures may also be justified with regard to such types of damage, even if the probability of their occurrence is uncertain or vague. This at least, provided the costs of the measures taken are reasonable.
  A further *variant* of this second position does not require precautionary measures to be taken if the possible benefits of an action are scientifically and plausibly weighted higher than any potential severe damage.

### The Epistemic Bases of Precautionary Decisions

A precautionary situation is one in which damage could occur but in which there is only limited knowledge about the probability of this possible damage occurring. The ethical idea of precaution, according to the thesis to be examined, justifies an obligation to take measures to prevent possible damage or to limit it to an extent not exceeding a permissible degree. This obligation exists even if no more is (yet) known about the probability of occurrence other than that it is greater than zero. Precautionary situations can therefore be seen as a particular type of risk situation. Decisions about precautionary situations are thus a type of risk decision.

Firstly, a distinction must be made between four types of epistemic basis on which risk decisions are made.

- It is known that damage will occur with 100% or 0% **certainty**: the damage is either sure to occur or sure not to occur. No statement of probability need be made.
- The damage scenario and its probability of occurrence are fully determinable. There is a situation of **complete or certain knowledge of the risk**. We know the statistical probability with which damage will occur. The risk is therefore calculable. In French and Italian specialist literature on the subject, this type of epistemic basis would be the object of *prevention*, not of precaution^14^.
- The damage scenarios are known. The bases on which their probability of occurring can be calculated are, however, imprecise to varying degrees. The probability of occurrence cannot therefore be calculated quantitatively but can only be estimated in qualitative terms. There is a situation of **incomplete or uncertain knowledge of the risk**. An example of this might be the exact prediction of avalanches: We know what the damage scenario is, but despite the various calculation models available, can only make a qualitative assessment of the probability of an avalanche occurring—as “high” or “low.”
- There are scientifically plausible indications for possible damage. Unlike type 3, however, it is not possible to estimate the probability of their occurrence. This epistemic situation is referred to below as **vagueness**. An example of such an epistemic situation of vagueness is the risk posed by a nuclear final storage facility. Owing to the time dimension, our geological and biological knowledge and experience are insufficient for us to make even a qualitative estimate of the probability of damage occurring.

To be distinguished from the four epistemic bases are situations of **ignorance**^15^. In such situations we do not know that we do not know. We have neither an idea of the damage potential nor do we have any (scientifically plausible) indications that give rise to fears. Therefore, there is no vagueness, but rather ignorance. A reaction is therefore impossible and there can thus be no obligation to take precautions. As soon as we have some form of hunch or fear, we are in a situation of uncertainty, no longer in a situation of ignorance.

It is important to bear in mind that uncertainty or vagueness refers only to the probability of occurrence, not to the damage scenarios. The damage is always known or at least there must be scientifically plausible indications of the damage scenarios. If the damage is not known or if there are no such indications, a situation of ignorance exists. Even complex situations do not mean that the damage scenarios are uncertain or doubtful, but rather that assessing their probability of occurrence becomes correspondingly more complex and therefore more difficult.

By the same token, epistemic uncertainty is to be distinguished from psychological uncertainty. If, based on a subjective assessment, someone fears that damage may occur and therefore feels insecure, this does not necessarily mean that there is epistemic uncertainty. There may be sufficient risk data to calculate the risk. Despite the psychological uncertainty, there would then be no epistemic uncertainty, but rather sufficient knowledge of the risk.

In practice, assigning a concrete decision situation to one of the theoretical types of epistemic basis regularly gives rise to controversy. Thus, it is debatable when a certainty of 100% or 0% can be assumed outside of controllable contexts, such as those that can be generated in a laboratory. When technologies are applied in the environment, there will always be a degree of uncertainty or vagueness. In the context of environmental risks, in particular, some people point to the complexity of the system and argue that such risk assessments are not only currently impossible, but that they are not feasible in principle. Others, on the other hand, assume that, even in complex systems, for certain types of events sufficient data may be available to determine the probability of occurrence or at least to provide a rough qualitative estimate. According to this position, even in the case of complex systems one should not therefore generally assume that a risk assessment is impossible.

These assignment issues and their role in precautionary decisions are discussed in section How Can an Ethical Decision be Made When Expert Opinions Differ? For the time being, it suffices to note that the precautionary idea relates to the epistemic bases of uncertainty and vagueness. Accordingly, measures are to be taken under the heading of precaution, although it is (still) uncertain or vague whether the feared damage will occur.

### How Do Ethics Theories Respond to the Epistemic Situation of Uncertainty?

What should be done when there is epistemic uncertainty and vagueness with regard to ethically relevant damage in the context of precaution? The answer to this question depends on the ethical theory of risk embraced.

Even if there are many competing ethical theories of risk, they can be assigned to only a limited number of types. Here we will focus on those two theory types which, according to a widely shared view, play the most dominant role in normative ethics, in general, as well as in (applied) attempts of answering the question of how to deal with precautionary situations: the consequentialist theories (the most well-known of which is the utilitarian theory) and the deontological theories^16^. These two theory types can be linked to all the environmental ethics positions mentioned in section What Constitutes Damage and Who or What Can Suffer Damage?

#### Deontological Ethics Theories

Common to all variants of deontological ethics theories is the notion that an action is morally right if it corresponds to the obligations that we have toward morally relevant entities. According to deontological ethics theories, entities are morally relevant because they have inherent value, i.e., value in themselves, regardless of their use or significance for others. Depending on the position taken, different entities have such inherent value: only humans or only living beings with certain characteristics, or all living beings or all collective entities. The obligations always exist toward the morally relevant individual entity with inherent value.

If there is a possibility that such an entity could suffer damage in an ethically relevant way, this would trigger a precautionary obligation. A precautionary obligation toward this entity does not rule out the possibility that measures must also be taken to protect other protection objectives, which do not have an inherent value. For example, if a precautionary obligation only exists toward people, this does not mean that no measures are to be taken to protect animals or environmental goods. The reason for these measures, however, lies not in the obligation toward these other beings or goods, but in the precautionary obligation toward the person for whom these beings or goods are of instrumental value.

Advocates of absolute deontological theories are obligated to refrain entirely, i.e., under all circumstances, from deeds that (could) damage entities with inherent value. Such absolute forms of deontological theory do not allow for any weighing up, even when there is a conflict of obligations. As inherent value cannot be weighted, making it impossible to calculate which obligation is of greater importance, in such cases advocates of deontological theories find themselves facing a dilemma. One variant of this approach excludes the weighing up of certain qualified goods only, such as human dignity, whereas for all other goods, a *prima facie* approach applies as described below.

Advocates of prima facie approaches of deontological risk theories permit a threshold value for damage, if it does not violate morally justified claims. They justify this by saying that an obligation to act always implies that it can also be fulfilled. An instruction that cannot be fulfilled is not plausible. If all action that could damage morally relevant entities were prohibited, life would not be possible, because with every action there is a probability that an entity with inherent value will be damaged. According to these prima facie approaches, exposing these entities to risks is reasonable if these risks are below the threshold value. If, on the other hand, they lie above the threshold value, measures should be taken to reduce the risks to below this value. If this is not possible, the action must be refrained from completely or at least until the risks can be reduced to below the threshold value. A special case of this variant of a threshold position assumes that, even below the threshold value, there is still an obligation to take further measures, insofar as they are proportionate.

In deontological risk theories, opportunities (i.e., more or less probable benefits) associated with an act may not be weighed against the associated risks^17^.

If complete risk knowledge is available, that is to say, it is known with which probability an entity with inherent value will be damaged by a certain action, advocates of deontological risk theories always decide according to the obligations that they have toward this entity. If the risk of being damaged is reasonable for the entity, the action is permissible. If the risk lies above the threshold value and is therefore unreasonable, the action must be refrained from.

If the risk knowledge is incomplete, the reasonableness and thus the admissibility of a risk cannot be determined. It is not known whether a certain action (or the application of a technology as a whole) exceeds the permitted threshold value. In such a situation, deontological approaches will require more data and information on the probability of damage occurring to morally relevant entities. The same is true to an even greater extent for situations in which there are only scientifically based theses that make serious damage appear plausible. In these cases, too, an obligation to carry out research may stem from this theory.

It should be borne in mind that risks must also be taken in order to obtain further risk information. These risks must also be reasonable. It follows from this that, according to deontological theories, this additional information can only be obtained gradually. This is the only way to obtain this information without exceeding the permitted risk threshold^18^.

#### Consequentialist Ethics Theories

There are also many types and variations of consequentialist ethics theories. The most well-known and politically influential is the utilitarian. It is therefore the focus of the following considerations. What all variants of this theoretical family have in common is that an action is assessed *solely* based on its consequences. For example, according to the act utilitarian theory, each action must maximize the expected net utility.

Because only the consequences of an action count, this precludes entities having inherent value in the deontological sense^19^. A change which is judged negative for a morally relevant entity according to deontological theory does not necessarily represent morally relevant damage according to utilitarian theory. Rather, it may be necessary to bring about such a change if it increases the net utility for all morally relevant entities. According to utilitarian theory, there would be morally relevant damage if an act did not increase this net utility.

If there is complete knowledge about opportunities and risks, these can be weighed up against each other and the best possible outcome for all ethically relevant entities can be calculated.

If the risk knowledge, i.e., the knowledge of opportunities and risks, is incomplete, further information is required according to consequentialist theories just as it is in the case of deontological theories, until it is possible to calculate the consequences (i.e., the net utility, according to the utilitarian theory). This is all the more the case when there are situations of vagueness in which there are only (scientifically founded) indications that serious damage may result.

In order to calculate the risk, information about both opportunities and risks for entities with moral value is required. New data is continuously considered in this calculation. Obtaining information also has its price^20^. In situations in which the opportunities are fully known, it may be that the price for additional risk information becomes so high that the calculation requires one to act without further risk information. However, a step-by-step approach must also be taken according to the logic of the consequentialist theories presented here. According to utilitarian theory, a step is taken when the calculation of the available information suggests that the net utility will be greater than if this step is not taken. As long as the data necessary for a calculation is unavailable, and the estimated cost of acquiring the data is not higher than the estimated opportunities, then there is a need for research.

### How Can an Ethical Decision Be Made When Expert Opinions Differ?

How do the different ethics theories react to a situation of disagreement or indecision about risk knowledge? If there is knowledge about possible damage, but the data on the probability of its occurrence is interpreted differently in expert circles^21^, advocates of both deontological and consequential risk theories will ask about the plausibility of the deviating interpretations. If the degree of plausibility of different interpretations varies, the more plausible expert opinion must be considered.

The degree of plausibility depends on the data available, the state of the art or the care taken in applying scientific methodology. Plausibility is decided based on the criteria for scientific excellence recognized by the scientific community: theory or hypothesis must, among other things, explain a particular phenomenon and be testable, meet coherence requirements and satisfy the principle of organized skepticism (e.g., undergo a peer review). A scientific hypothesis is thus considered plausible if there is much to be said for its correctness. This is, so to speak, the threshold that separates plausibility from non-plausibility.

It is the task of the scientific community to assess scientific plausibility. In order to fulfill this task according to scientific criteria, the institutions need access to the information that led to the formulation of the scientific theses. The data must be presented in a comprehensible manner, including data that does *not* support the scientific thesis. Furthermore, the scientific institutions must be independent to ensure that plausibility is assessed impartially and according to scientific criteria.

What should be done when disagreement or indecision still exists within the scientific community and the question of plausibility cannot be decided in a scientific manner? If there are two or more competing positions that all meet the plausibility criteria and have large groups of advocates in the scientific community, it is usually also accepted within the community that there is a state of disagreement. From an ethical standpoint, therefore, research is required. More information is required to find out which of the interpretations is more plausible.

If, on the other hand, a large majority of the scientific community considers the data situation to be clear, the role of a deviating minority opinion must be examined nevertheless. Must the majority opinion be followed or is there a situation of scientific uncertainty? First of all, it should be noted that neither the fact that a scientific position is held by a majority nor by a minority is a criterion for its correctness. Even when everyone agrees, this does not mean the position is true *for this reason*. Conversely, the plausibility of a position cannot be determined independently of the sciences. If this were possible, it would be possible to make an objective and unequivocal decision on which theories are plausible based on criteria independent of science. It is conceivable that there are several plausible theses concerning the same facts or phenomena. Theoretically, it should be possible to use plausibility criteria to decide which of the gradually differing plausible positions is the most plausible. In practice, however, the scientific community is generally unable to judge so easily either the question of plausibility or the question of the degree of plausibility.

Nevertheless, in such undecided and indecisive situations, decisions have to be made. For this reason, it is imperative that decision-makers, such as public authorities, check whether the criteria for scientific research have been adhered to, and to what extent competing positions are plausible, in order to be able to understand the assessments of the scientific institutions and classify them appropriately. They therefore also require access to the necessary information in a comprehensible form, including diverging data that does not support the scientific theses. These authorities should therefore also have employees with this kind of scientific training. It is not their responsibility to carry out a plausibility assessment themselves, but they must be able to critically understand those made by the scientific community. It should be noted that these employees act as representatives of the political decision-making authorities and thus play a role different to that of the academic institutions.

### Different Theories, Converging Practices

There are different approaches to justifying the concept of precaution depending on the ethical theory of risk. Nevertheless, if there are indicators of a precautionary situation, and if the criteria that trigger measures are met, advocates of both deontological and consequentialist theories largely agree over the implications of the precautionary measures and the form that they should take. They agree on this in spite of all their theoretical differences, including the relevance they assign to the consequences which new technologies may have. According to both risk theories, there is an obligation to act in a precautionary manner. Both demand an obligation to obtain comprehensive information in order to reduce uncertainties with the aim of enabling suitable risk assessment.

## Precautionary Obligations

Precautionary situations differ from other risk situations in that, firstly, *serious damage* is possible and secondly, the *probability of occurrence* is *epistemologically uncertain*. If both these criteria are met, there is an ethical obligation to take precautionary measures. Precautionary measures can and must be taken, therefore, if the existence of the two criteria is established. There are two conceivable options:

- According to the *first option*, those who fear that serious damage may occur must show that their fear lies within a plausible range.
- According to the *second option*, the burden of truth is reversed. It is not up to those who fear the occurrence of serious damage to demonstrate plausible grounds for this fear. Rather, it is the responsibility of those whose actions give rise to the fear of serious damage occurring to demonstrate plausibly why such damage is extremely improbable or scientifically absurd.

If there are plausible indications of serious damage, the reversal of the burden of truth is justified. Furthermore, in precautionary situations, i.e., in situations in which it is feared that possibly serious damage may occur, the obligation to ensure that precautionary measures are taken lies primarily with the state authorities responsible for safeguarding the protection objectives in question.

The issue of how to apply new (bio) technologies in the environment and identify the role of precaution in this context is more than a purely legal or scientific one. Owing to the far-reaching consequences of these technologies, such as the (unintended) rise of new and unknown animal and human diseases or the reduction of biodiversity in the attempt to combat malaria using CRISPR/Cas-based gene drives, not only are the state authorities called upon, but the answers must be negotiated by society in the political process. The decision whether to use genome editing to fight malaria in endemic areas, by way of example, neither belongs solely to science nor to legal authorities, but also requires engagement of the local communities who are particularly prone to foreign economic interests^22^. While the state is solely responsible for the political decision-making processes^23^, this is not inconsistent with the fact that the public authorities rely on the involvement of others^24^ in order to fulfill their responsibilities.

Various instruments of precaution are conceivable considering both the political decision-making processes and actual proposals for regulations. No attempt is made here to provide a definitive list of these instruments.

Taking precautionary measures in favor of protection objectives or ensuring that an ethically unjustifiable occurrence of damage is (highly) unlikely often involves prohibiting or refraining from an activity or a certain application, and thus raises the question of if, and to what extent, precaution can be ethically reconciled with basic and more specific freedom rights. Arguably, the answer to this question depends on a more in-depth account of the moral value of precaution, of freedom, and of their relation, which goes beyond the scope of this paper. However, restricting freedom rights in some way may be justified if the measures taken are proportionate with regard to the protection of freedom rights. If, for example, plausible fears exist, but owing to a lack of knowledge or unanimity about the knowledge available it is still unclear whether these fears will continue to be justified in the future, the appropriate measure is not a general prohibition, but a temporary one (moratorium). Furthermore, rather than general prohibition, spatial or application-specific prohibition should be considered.

However, there is a need to counter the frequently expressed reservation that precautionary measures necessarily only involve proscription. Precautionary measures can also exist as *orders* to act. The obligation to proceed step by step, for example, means that missing knowledge can be acquired and potential serious damage restricted at an early stage. When the first astronauts landed on the Moon, it was feared that they might bring back microbes, which could lead to catastrophic effects on earth. This fear, which was plausible relative to the state of knowledge at that time, did not mean that the moon landing was prohibited. Instead, the astronauts had to spend 3 months in quarantine upon their return, a precautionary measure that effectively assuaged the fears.

Besides the state agencies responsible for determining precautionary situations and for the binding definition of measures, other players also have a moral duty. These might be companies and manufacturers that produce potentially harmful substances or that introduce them into the environment as well as agricultural holdings. Businesses and manufacturers have the duty to work with such substances in accordance with the regulations and rules of good professional practice. The idea of precaution also obliges them to report any unexpected adverse effects noticed, so that appropriate precautions can be taken. As a result, the state also has a duty to create agencies to which such observations can be reported, and to react in good time.

Research scientists and research institutions also have a responsibility, as they are often the first or the only ones able to recognize the damage potential of their research activities. They have a duty to work in compliance with the rules set within their scientific field, and to take precautionary measures to avoid serious damage in the context of their research activities. This may mean that precautionary measures are already called for when research projects are appraised or funded, if scientifically plausible damage scenarios are apparent. For example, state research funding may not be one-sided and a range of research prospects and research paradigms should be considered. Furthermore, researchers and research institutions are required to draw the attention of the authorities and the public at an early stage to developments which may have precautionary relevance. Here also, it is the state's duty to receive such information and to react expeditiously.

If all the players involved are to be able to observe their precautionary obligations, the responsible actors in the education system are also called upon. Pupils and students should be made aware of the issues in a way appropriate to their level of competence, and taught how to deal with knowledge, uncertainty and risk situations. This should happen above all at tertiary level, i.e., in universities, and in vocational training for occupations, which are confronted with such precautionary situations. In the context of biotechnology, this includes agricultural colleges.

Dealing with new technologies in the environment does not only affect those in the research field or those who apply these technologies in their work. Because of their potential impact, how to deal with new technologies in the environment and the extent to which it is permissible to expose third parties to risks are issues of importance to the whole of society. In Switzerland, therefore, they are regularly the subject of political popular votes.

## Recommendations

1. **Consistent strengthening and application of the idea of precaution**. With regard to new biotechnologies, the applicability of the legal concept of precaution is frequently questioned. However, the idea of precaution can also be legitimized ethically, irrespective of the underlying ethical risk theories. This leads to the first recommendation, namely to adhere to the concept of precaution in the regulation of new biotechnologies,^25^ to establish it firmly in the further development of environmental law and to support its application at international level.
  The question of how to deal with epistemic uncertainties and thus with precautionary situations is closely related to the question of how we generate knowledge. It also affects the political culture in which we make decisions involving technologies and uncertainty. The following recommendations therefore relate to the conditions under which knowledge is acquired and political decisions are made.
2. **Improving the reliability of risk assessments**. The data on which a risk analysis is based must satisfy scientific criteria. It is the responsibility of the scientific institutions to comply with these criteria, and they have their own mechanisms for doing so. The framework conditions of the scientific institutions should be strengthened in such a way that they are able to meet the criteria in a scientifically independent manner and can consistently demand that all actors comply with the scientific standards and justification requirements. Scientific data and assessments must also be verifiable and comprehensible in order to meet internal scientific controls, and thus satisfy scientific criteria. This involves granting access to all information necessary for scientific evaluation, including to divergent data that does *not* support a scientific thesis^26^. Furthermore, attention must be paid to promoting and cultivating diversity of perspectives and cross-sectional competences.
  Access to data and transparency of scientific assessments are also essential for decision-making authorities; they must be able to understand the plausibility of scientific data and how they have been assessed, in order to be able to make reasoned decisions. Moreover, they must be able to present the risk-related decisions that affect the public in a transparent and understandable manner.
  This is the only way to ensure that voters can form free and informed opinions, and thus that risk decisions in the political process can be reliable.
3. **Respecting the different roles of expert committees, on the one hand, and of decision-making authorities and the courts, on the other**. Decisions about dealing with new (bio)technologies in the environment have far-reaching consequences, which are of relevance to the whole of society. The decisions may therefore not be left to individuals, nor may the democratically legitimate authorities in charge of making these decisions delegate them to others.
  This also means that decision-making within specialized bodies advising the competent authorities must be subject to democratic control. This requires their decision-making process to be transparent and comprehensible, and majority opinions and minority positions must be presented openly and comprehensibly with justifications. Furthermore, given both the plurality of scientific opinions and the fact that the state may not delegate decisions in such matters, it follows that neither the decision-making authorities nor jurisdiction automatically accept the expert opinions of specialized advisory bodies. The decision-making authorities must therefore also have appropriately trained staff capable of critically following the plausibility checks and assessments made by the scientific institutions.
4. **Strengthening political awareness in dealing with technologies and uncertainties**. Decisions on how to deal with technologies involve uncertainties and possibly have far-reaching consequences. The decisions are based on risk assessments that involve making decisions about values. In democratic societies, the responsibility for these value decisions lies with the citizens, not with scientists. Awareness of this fact must also be raised among the employees of authorities who implement such value decisions when assessing individual cases. If they are involved in this decision-making process as specialists, they do so on behalf of the political authority. Their role as scientists in this context is thus different from that of their colleagues in scientific institutions.

## Conclusion

The rapid development of new biotechnologies such as CRISPR-Cas systems and other genome editing processes opens up new opportunities and promises a wide range of applications, although it is yet to be seen whether all this potential can be realized. At the same time, the new technologies and their application potential confront us with considerable uncertainties. On the one hand, we do not know everything about how the new technologies function or about their impact to organisms on which they are applied. If the technologies and organisms, which have been altered by the processes, come into contact with the environment, this not only increases the complexity of possible interactions, but also our uncertainties.

Environmental law responds to this epistemic situation of uncertainty with the legal concept of the precautionary principle or precautionary approach. If serious damage is not merely conceivable, but there is also a scientifically plausible foundation for the fear that such damage could occur, then a precautionary obligation exists. It is concluded that the concept of precaution in environmental law and the precautionary measures to which it gives rise can also be justified ethically, irrespectively of the underlying ethical theory of risk.

## Author Contributions

All authors listed have made a substantial, direct and intellectual contribution to the work, and approved it for publication.

### Conflict of Interest Statement

The authors declare that the research was conducted in the absence of any commercial or financial relationships that could be construed as a potential conflict of interest.

## Footnotes

^1^The so-called CRISPR/Cas systems are among the genome editing methods currently under discussion. They allow the targeted modification, insertion or removal of individual DNA building blocks. The method was scientifically documented for the first time in 2012 and can be applied to almost all organisms.

^2^In organisms with sexual reproduction, a gene drive is the (naturally occurring or engineered) mechanism by which particular genes or suites of genes and the corresponding phenotypes are propagated throughout a population with a chance greated than (the normal, i.e., Mendelian) 50%.

^3^This paper is based on a report of the Swiss Federal Ethics Committee on Non-Human Biotechnology (ECNH). The report was published in May 2018 (http://www.ekah.admin.ch/en/ecnh-opinions-and-reports/ecnh-reports/). Its current members, elected by the Federal Council for a four-year legislature, are Markus Arnold, Monika Betzler, Christine Clavien, Eva Gelinsky, Greta Guarda, Gérald Hess, Tosso Leeb, Matthias Mahlmann, Jean-Marc Neuhaus, Klaus Peter Rippe, Otto Schäfer, and Markus Wild. The authors would like to thank Andreas Bachmann for his critical inputs throughout the process of writing as well as Nina Scherrer for her support concerning literature research.

^4^http://www.un.org/documents/ga/conf151/aconf15126-1annex1.htm

^5^http://eur-lex.europa.eu/legal-content/EN/TXT/PDF/?uri=CELEX:52000DC0001&from=EN

^6^Directive 2001/18/EC of the European Parliament and of the Council of 12 March 2001 on the deliberate release into the environment of genetically modified organisms and repealing Council Directive 90/220/EEC (https://eur-lex.europa.eu/eli/dir/2001/18/oj); Cartagena Protocol on Biosafety (https://bch.cbd.int/protocol/text/)

^7^Swiss Federal Constitution, Article 74 (https://www.admin.ch/opc/en/classified-compilation/19995395/index.html)

^8^Swiss Federal Act on the Protection of the Environment, Articles 1 and 11 (https://www.admin.ch/opc/en/classified-compilation/19830267/index.html)

^9^Swiss Federal Act on Non-Human Gene Technology, Article 2 (https://www.admin.ch/opc/en/classified-compilation/19996136/index.html)

^10^The criterion of restricted knowledge could perhaps be construed from the formulation that measures shall be taken *early*. This would have to mean that action should be taken not at the time when imminent danger is to be averted, but earlier, when there is no certainty as to the probability of the damage or nuisance occurring.

^11^Only specialist language, in other words, aims at capturing the shift from a (preventive) approach in which “the decision-maker intervenes provided that the threats to the environment are tangible” to a (precautionary) approach under which “authorities are prepared to tackle risks for which there is no definitive proof that there is a link of causation between the suspected activity and the harm or whether the suspected damage will materialize” (de Sadeleer, 2010).

^12^Cf. ECNH, Dignity of Living Beings with regard to Plants. Moral consideration of plants for their own sake, 2008, p. 19, and ECNH, Ethical Treatment of Fish, 2014, p. 21 f, including criticism of the different positions

^13^In terms of precaution, the Rio Declaration talks not only of possible *serious*, but of *irreversible* damage. Any change is, essentially, irreversible. If, for example, a forest is destroyed, it is not possible to restore it to exactly the same form, even if reforestation takes place over a long period. The living creatures that formed part of the forest cannot be brought back. The forest is a new forest with new living creatures. In an ecological context, however, the concept of irreversibility is not usually understood in this way. A forest that can be restored, or a particular moth which disappears but of which examples of the same species become re-established, are not considered to have been lost irreversibly. According to this understanding of irreversibility, the damage can be compensated. The term is used to qualify a particular type of damage: damage that has long-term effects and affects things that are considered particularly important and valuable to the human community (possibly also to later generations) and its environment. Understood thus, irreversibility serves as an indicator when assessing how serious any damage caused may be, but not as an independent criterion for precautionary measures.

^14^See also section Precaution and Prevention.

^15^Others deny the relevance of the distinction between uncertainty and risk by arguing that, practically, uncertainty is a case of risk (cf. Roser, 2017).

^16^We note that, besides consequentialist and deontological approaches, virtue ethical accounts—which focus not so much on consequences nor on obligations but emphasize the virtues or (moral) character of the (moral) agent—are often considered a genuine, i.e., irreducible, third alternative. Here, however, as we focus on the most basic, underlying logic of competing ethical theories (of risk), we assume that virtue ethical aspects can ultimately be assigned to either consequentialist or deontological types of considerations.

^17^There is disagreement among advocates of deontological ethics over whether opportunities that enable the fulfillment of positive obligations should be taken into account.

^18^It remains to be seen how these threshold values are to be set and how one knows when enough information is available in order to establish when the risk is no longer reasonable.

^19^For advocates of a utilitarian theory, the individual being or individual entity never has value for its own sake.

^20^See: Christian Munthe, Precaution and Ethics. Handling risks, uncertainties and knowledge gaps in the regulation of new biotechnologies, Report commissioned by the ECNH, published as Volume 12 of the ECNH publication series “Contributions to Ethics and Biotechnology”, 2017.

^21^There are many reasons why scientific results are interpreted in a variety of ways. Scientific disagreement often results from ambiguous and inaccurately positive results of research. There is a further problem with interpreting data when studies do not meet statistical relevance requirements. This makes it even more important to create transparency about the basic assumptions on which scientific interpretations are based.

^22^cf. Patrão Neves and Druml (2017) a report on the UNESCO Chair of Bioethics' meeting at the Medical University of Vienna in September 2016, which gathered infectious disease experts with a focus on malaria, entomologists and ethicists to discuss the advantages and disadvantages of genome editing applied to mosquitoes to fight malaria.

^23^A further option is theoretically conceivable, namely that a precautionary situation can always be assumed, i.e. that it is always clear that the criteria are met. Such a position, which means a general reversal of the burden of proof for all actions, would however encroach on freedom rights to a disproportionate degree and cannot therefore be ethically justified.

^24^Wareham and Nardini (2015), for example, propose a method of public deliberation to discriminate negligible from non-negligible risks with respect to the application of synthetic biotechnology.

^25^cf. the current legal discussion in Switzerland, in which adherence to the precautionary principle is recommended (Errass, 2018).

^26^In view of recent developments in science and education policy, care must be taken to ensure that conflicts of interest do not restrict impartial research at universities. Such restrictions not only compromise the independence of scientists but also alter the self-conception of scientific institutions. They may affect the quality of scientific data, influence the choice of research approaches and, at worst, lead to interest-based solutions and results. In all cases, such restrictions undermine confidence in the independence of science and the scientific quality of data and data assessment.
